# Deafness

**Published:** 1854-07

**Authors:** 


Deafness.—The *Perthshire Advertiser* reports a recently discovered mode of conversing with those afflicted with partia deafness, viz., by taking the individual by the hand, at the same time placing the two thumbs together. By this simple process, the sound is conveyed in a more direct manner to the ear, and the person spoken to will hear distinctly in a tone of voice several notes lower. It is also important to add, that a chain could be formed upon the same principle, by a number joining hands in the manner alluded to, when the individual affected will hear in a moderate key at either end of the chain.

